# Wireless Sensor Technologies and Applications

**DOI:** 10.3390/s91108824

**Published:** 2009-11-04

**Authors:** Feng Xia

**Affiliations:** School of Software, Dalian University of Technology, Dalian 116620, China; E-Mail: f.xia@ieee.org

Recent years have witnessed tremendous advances in the design and applications of wirelessly networked and embedded sensors. Wireless sensor nodes are typically low-cost, low-power, small devices equipped with limited sensing, data processing and wireless communication capabilities, as well as power supplies. They leverage the concept of wireless sensor networks (WSNs), in which a large (possibly huge) number of collaborative sensor nodes could be deployed. As an outcome of the convergence of micro-electro-mechanical systems (MEMS) technology, wireless communications, and digital electronics, WSNs represent a significant improvement over traditional sensors. In fact, the rapid evolution of WSN technology has accelerated the development and deployment of various novel types of wireless sensors, e.g., multimedia sensors. Fulfilling Moore's law, wireless sensors are becoming smaller and cheaper, and at the same time more powerful and ubiquitous.

As shown in Figure 1, there are typically four main components in a sensor node [1], i.e., a sensing unit, a processing unit, a communication unit, and power supply. The sensing unit may be composed of one or more sensors and Analog-to-Digital Converters (ADCs). Sensors are hardware devices that measure some physical data of the monitored system's state such as temperature, humidity, pressure, or speed. The analog signals produced by the sensors are digitized by ADCs and sent to the processing unit for further processing. Within the processing unit, there is a microcontroller associated with a small storage unit including on-chip memory and flash memory. The processing unit is responsible for performing tasks, processing data, and controlling the functionality of other components of the sensor node. A wireless sensor connects with other nodes via the communication unit, where a transceiver encompasses the functionality of both transmitter and receiver. The wireless transmission media may be radio frequency, optical (laser), or infrared. At present, the main type of power supply for wireless sensor node are sbatteries, either rechargeable or non-rechargeable. Energy is consumed for sensing, data processing, and communication. For small wireless sensor nodes (with limited computing capacity), data communication will expend the majority of energy, while sensing and data processing are much less energy-consuming.

In the past one and a half decades, a number of prototype and commercial wireless sensor nodes have been made available by research institutions and companies from around the world. Although these sensor nodes often differ in capacity and feature, most (if not all) of them have been built upon the architecture given in Figure 1. Table 1 gives a list of some available wireless sensor nodes.

The proliferation of these products opens up unprecedented opportunities for a wide variety of scientific, industrial, agricultural, commercial and military applications, such as health care, smart transportation, emergency response, home automation, social studies, critical infrastructure protection, and target tracking, just to mention a few. In particular, wireless sensor and actuator networks are a key enabling technology for cyber-physical systems [2,3], which will ultimately improve the quality of our lives. To realize the full potential of wireless sensors, enormous challenges need to be addressed and significant efforts have been made in this field.

## In This Issue

The objective of this Special Issue was to gather the latest research and development achievements in the field of wireless sensors and to promote their real world applications. Special attention is paid to several important aspects of wireless sensor technologies, i.e., sensor networking, localization, and power management, as well as design, implementation, and applications of wireless sensors. The issue includes a total of 46 high-quality papers, which are expected to give the readers some insight into the current state of the art

A considerable portion of these papers deal with diverse issues in sensor networking. Qiu *et al.* [4] introduce a unified multi-functional dynamic spectrum access framework. Jung and Park [5] propose a cache-based sensor network bridge, which enables sensing data reusability and customized WSN services. Hung *et al.* [6] present an energy-efficient secure routing and key management scheme for mobile sinks in sensor networks. Availability and end-to-end reliability in low duty cycle multi-hop WSNs are addressed by Suhonen *et al.* in [7]. A MAC-aware data aggregation method is proposed in [8] by Li and co-workers to minimize the total energy consumption of data transmission. Qiu *et al.* [9] propose the priority-based coverage-aware congestion control algorithm which is distributed, priority-distinct, and fair. Amin *et al.* [10] design a robust intrusion detection system for IP-based sensor networks. Son *et al.* [11] study the problem of how to alleviate the exposed terminal effect in multihop wireless networks in the presence of log-normal shadowing channels. Other topics examined include distributed joint source-channel coding [12], network coverage [13,14], sensor deployment [15,16], fault detection [17], and security [18-20]. Some important aspects of WSNs are reviewed in [21] and [22].

The knowledge of position is indispensable for many applications and services provided by WSNs. Teng *et al.* [23] introduce a range-free, distributed and probabilistic mobile beacon-assisted localization approach for static WSNs. They also present an improved version of the approach. Pei *et al.* [24] propose an anchor-free localization method for mobile targets based on non-metric multi-dimensional scaling and rank sequence. A network-based mobility scheme for mobile 6LoWPAN nodes is presented by Bag *et al* [25]. Lloret *et al.* [26] propose a hybrid stochastic approach to self-location of wireless sensors in indoor environments. Jeon *et al.* [27] propose a sink-oriented dynamic location service for handling sink mobility.

Saving energy is of paramount importance for wireless sensors. Knight *et al.* [28] review the state-of-the art technology in the field of both energy storage and energy harvesting for sensor nodes. Priya *et al.* [29] review the progress made in the synthesis of thick film-based piezoelectric and magnetoelectric structures for harvesting energy from mechanical vibrations and magnetic field. The problem of sensor scheduling with a mobile sink is studied by Maheswararajah *et al.* [30], with focus on minimizing the total energy consumed by sensor nodes while avoiding measurement losses. Two sleep scheduling management schemes for WSNs are presented in [31]. In [32], high-resolution images with a wide field of view are generated with minimum energy dissipation. An adjacency matrix-based transmit power control method is presented by Consolini *et al.* in [33].

Several papers are about the design of application-oriented sensors. In [34] Wang *et al.* develop a passive wireless temperature sensor, capable of working in harsh environments and suitable for monitoring high temperature rotating components. A wireless sensor node for precision horticulture which permits the use of precision agricultural instruments based on the SDI-12 standard is developed in [35]. Rodrigues *et al.* [36] present the design and implementation of an intra-body sensor for acquisition and monitoring of intra-vaginal temperatures. Bartolozzi and Indiveri [37] present a neuromorphic VLSI device, i.e., the Selective Attention Chip, which can be used in multi-chip address-event systems.

Sensor-based applications have been reported in a number of papers. Jurdak *et al.* [38] propose to integrate sensor networks with medium range wireless mesh networks to realize large scale environmental monitoring. Song *et al.* [39] develop a mobile sensor network system for monitoring applications in unfriendly environments. Key technologies for wireless monitoring of intelligent automobile tires are discussed in [40]. Wang and Niu [41] propose a method for spatial forecast of landslides in Three Gorges using the spatial data mining technology. Raza *et al.* [42] present a web portal framework for sensor-based applications in pervasive computing environments. Zhang *et al.* [43] introduce a two-stage approach to the detection of people eating and/or drinking for the purpose of living surveillance. The design and evaluation of a WSN based aircraft strength testing system is reported in [44]. Water monitoring using wireless sensors is reported in [45]. Handcock *et al.* [46] realize the monitoring of animal behaviour and environmental interactions using ground-based sensors, GPS collars and satellite remote sensing. The relevance of using open hardware and software motes for environment monitoring is assessed by Bagula *et al* [47]. Antoine-Santoni *et al.* [48] deal with a WSN as a reliable solution for capturing the kinematics of a fire front spreading over a fuel bed. Wireless sensor technologies and applications in agriculture and food industry are reviewed in [49].

It is my hope that the readers would find this Special Issue interesting and useful in their research and development work. I would like to express my whole-hearted thanks to all the authors who have submitted their papers to this issue. I am also very grateful to all the reviewers for their valuable comments and suggestions that guarantee the quality of the papers published. Finally, I want to thank Dr. Ophelia Han, Mr. Dietrich Rordorf, Mr. Matthias Burkhalter, Dr. Shu-Kun Lin and their staff at the Sensors Editorial Office for their great support and the opportunity to run this Special Issue.

## Figures and Tables

**Figure 1.**
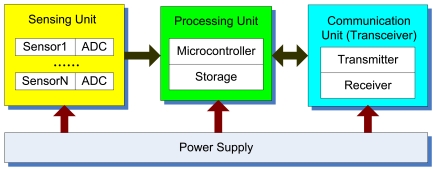
Wireless sensor architecture.

**Table 1.** Some available wireless sensor nodes.

**Node**	**Sensing Unit**	**Microcontroller**	**Memory**	**Transceiver**
BTnode	UART, SPI, I2C, GPIO, ADC, etc	ATmega 128L	4KB EEPROM, 64KB SRAM, 128KB FLASH	Chipcon CC1000; Zeevo ZV4002 Bluetooth
FireFly	Sensor expansion card: temperature, light, acoustic, etc	ATmega 1281	8KB RAM, 128KB ROM	Chipcon CC2420
IMote2	UART, SPI, I2C, SDIO, GPIO, etc	Intel PXA271	256KB SRAM, 32MB FLASH, 32MB SDRAM	CC2420
MicaZ	Expansion connector for light, pressure, acceleration, etc	ATmega 128L	4KB RAM, 128KB FLASH	CC2420
SunSPOT	Temperature, light, acceleration, etc	ARM 920T	512KB RAM, 4MB FLASH	CC2420
TinyNode584	On-board temperature sensor	TI MSP430	10KB SRAM, 48KB FLASH	Xemics XE1205
Tmote Sky	On-board humidity, temperature and light sensors	TI MSP430	10KB RAM, 48KB FLASH	CC2420
